# Computational analysis of protein-protein interaction network of differentially expressed genes in benign prostatic hyperplasia

**DOI:** 10.22099/mbrc.2022.43721.1746

**Published:** 2022-06

**Authors:** Ruchi Sachdeva, Navneet Kaur, Paras Kapoor, Pooja Singla, Nidhi Thakur, Sakshi Singhmar

**Affiliations:** Department of Bioinformatics, Goswami Ganesh Dutta Sanatan Dharma College, Sector-32C, Chandigarh, India

**Keywords:** Prostate enlargement, lower urinary tract symptoms, microarray, gene expression, cancer, therapeutic drug target

## Abstract

Benign prostatic hyperplasia (BPH) is a commonly occurring disease in aging men. It involves cellular proliferation of stromal and glandular tissues leading to prostate enlargement. Current drug therapies show several adverse effects such as sexual dysfunctions and cardiovascular side effects. Therefore, there is a need to develop more effective medical treatment for BPH. In this regard, we aimed to identify genes which play a critical role in BPH. We have obtained the dataset of differentially expressed genes (DEGs) of BPH from NCBI GEO. DEGs were investigated in the context of their protein-protein interactions (PPI). Hub genes i.e. genes associated with BPH were scrutinized based on the topological parameters of the PPI network. These were analyzed for functional annotations, pathway enrichment analysis and transcriptional regulation. In total, 38 hub genes were identified. Hub genes such as transcription factor activator protein-1 and adiponectin were found to play key roles in cellular proliferation and inflammation. Another gene peroxisome proliferator activated receptor gamma was suggested to cause obesity, a common comorbidity of BPH. Moreover, our results indicated an important role of transforming growth factor-beta (TGF-β) signaling and smooth muscle cell proliferation which may be responsible for prostate overgrowth and associated lower urinary tract symptoms frequently encountered in BPH patients. Zinc finger protein Snai1 was the most prominent transcription factor regulating the expression of hub genes that participate in TGF-β signaling. Overall, our study has revealed significant hub genes that can be employed as drug targets to develop potential therapeutic interventions to treat BPH.

## INTRODUCTION

Benign prostatic hyperplasia (BPH), a common health concern in aging men, is characterized by enlargement of the prostate gland. Its incidence varies with age and usually it begins at the age of 40-45 years [1]. Prevalence of BPH is 5-10% in men aged 40, but increases to 80% in men of age group 70-80 years [2]. BPH is accompanied by inflammation in the prostate gland. It also manifests increased prostate volume which is mainly caused due to the cellular proliferation occurring in its transition zone that surrounds the urethra. This results in pushing on the urethra leading to the development of irritating and obstructive lower urinary tract symptoms (LUTS) [3, 4]. LUTS mainly involves two types of symptoms - voiding symptoms include weak urinary stream, stop-start urination, a sensation of incomplete emptying of bladder, and storage symptoms such as frequency, nocturia, and urgency [5]. These symptoms worsen with age and are extremely bothersome, thereby affecting the quality of life.

Current drug therapies work by targeting the proliferative action of androgens leading to decrease in the size of the prostate. This includes drugs which block the activity of 5α-reductase enzyme that converts testosterone to a more potent androgen. Other BPH treatments include the use of drugs which decrease the tone of the smooth muscle by inhibiting α1 adrenoceptors [6]. Although these strategies are able to relieve urethral obstruction and voiding symptoms, they are associated with some side effects including abnormal ejaculatory effects. Alternative treatment options explore the use of novel drug targets such as luteinizing hormone releasing hormone (LHRH) receptor, muscarinic receptor, phosphodiesterase among others [7, 8]. However, results from clinical trials on drugs targeting these emerging drug targets indicate that they may cause sexual dysfunctions [9]. Thus, despite the availability of drug targets against BPH, there is still a need to investigate novel proteins that can be employed for developing better medical treatments.

In the past decade, protein-protein interaction (PPI) network has gained a lot of importance in the search of therapeutic drug targets [10, 11]. PPI networks help to study proteins from the perspective of their relationships and associations with their functional partners. Thus, computational analysis of PPI networks provides novel insights into the functions of proteins and their role in several biological processes. So considering the urgent need of promising therapeutic interventions to treat BPH, we made an attempt to predict potential drug targets based on the knowledge of protein-protein interactions of genes related to the disease.

## MATERIALS AND METHODS

**Gene expression data retrieval:** The gene expression dataset for Benign prostatic hyperplasia was obtained from the gene expression omnibus (GEO) database under the series accession number GSE119195 [12]. This dataset consisted of expression profiles of prostate tissues from 3 healthy individuals and 5 BPH patients. In this study, microarray analysis of mRNA isolated from prostate tissues was performed according to the standard Affymetrix protocol followed by data analysis and normalization [12].

Firstly, raw CEL files under the GEO accession number GSE119195 were downloaded. The data was analyzed using GEO2R (http://www.ncbi.nlm.nih.gov/geo/geo2r/) which is an interactive web tool used for comparing different GEO series in order to find genes that are differentially expressed across experimental conditions. The genes were initially filtered according to their significance by taking the criteria of P-value being less than 0.05. Finally, differentially expressed genes were identified based on log_2_Fold Change [log_2_(FC)] value. Genes with log_2_(FC) value>1 were considered as up-regulated that indicated 2-fold greater expression in diseased tissue as compared to the healthy one. While those having log_2_(FC) value <-1 were filtered as down-regulated genes, i.e. having 2-fold lower expression.

**Protein-Protein interactions analysis:** The resultant differentially expressed genes were uploaded to STRING version 11.0b database (Search Tool for the Retrieval of Interacting Genes/Proteins) in order to find their interactions with other proteins. STRING provides the protein-protein interactions including both physical and functional associations derived from primary databases, high throughput lab experiments, computational prediction, conserved co-expression analysis and automated text mining of the scientific literature [13]. Only the high confidence interactions having score>0.7 were retrieved. Taking these genes further, protein-protein interaction (PPI) network was constructed and visualized with the help of Cytoscape [14].

**Analysis of PPI** **network:** The PPI network was analyzed using NetworkAnalyzer which is an in-built tool of the Cytoscape version 3.8.2. The analysis was done by calculating topological parameters like node degree, closeness centrality and betweenness centrality. Node degree (or simply degree) represents the number of interactions of a node to other nodes in the network. Closeness centrality measures the average shortest distance between one node and all other nodes in the network. This indicates that the most central nodes are closer to other nodes in the network. Betweenness centrality is measured by adding lengths of all the shortest paths that pass through a node. So, nodes having high betweenness centrality serve as a bridge that connects other nodes. Thus, closeness centrality and betweenness centrality are two crucial centrality measures that shed light on the significance of a node for the information flow through the network. Based on these parameters, the most important genes designated as hub genes were identified.

**Co-expression analysis of the hub genes** **: **The hub genes so obtained were cross-checked by the FpClass tool that uses topology network score and gene expression score in order to find the genes coexpressed along with the retrieved genes. The topology network score indicates the presence of genes in the data and the intensity of their interactions while the gene expression score takes into account the correlation of the expression profiles of genes [15]. The genes with topology score>= 0.75 for up-regulated and>=0.7 for down-regulated were selected for further analysis. 

**Functional enrichment and pathway analysis:** The hub genes were examined to determine their biological functions and roles in pathways. This was done using ClueGO, a plug-in application of Cytoscape [16]. ClueGo uses both Gene Ontology (GO) terms and the Kyoto Encyclopedia of Genes and Genomes (KEGG)/BioCarta pathways to create a functionally enriched pathway network. The localization of the biological and molecular functions of the genes was detected premised on high confidence with the p-value kept <0.05.

**Identification of transcription factors and miRNA associated with BPH:** The transcriptional factors (TF) for the hub genes were determined using the MatInspector tool [17]. The top 7 transcriptional factors having p-values>0.1 were taken. Finally, the miRNAs for selected transcriptional factors were determined with the help of miRTargetLink [18]. The common miRNAs for the TFs were filtered. These TFs and miRNAs were integrated and an interactive network was plotted in Cytoscape.

## RESULTS

The gene expression dataset for healthy individuals and BPH patients were retrieved from the GEO NCBI database. After data normalization and filtering based on P-value and log_2_(FC) value, a total of 338 DEGs were found. Among them, 200 genes with log_2_(FC) value <-1 were identified as down-regulated and 118 genes with log_2_(FC) value>1 as up-regulated.

**Figure 1:**
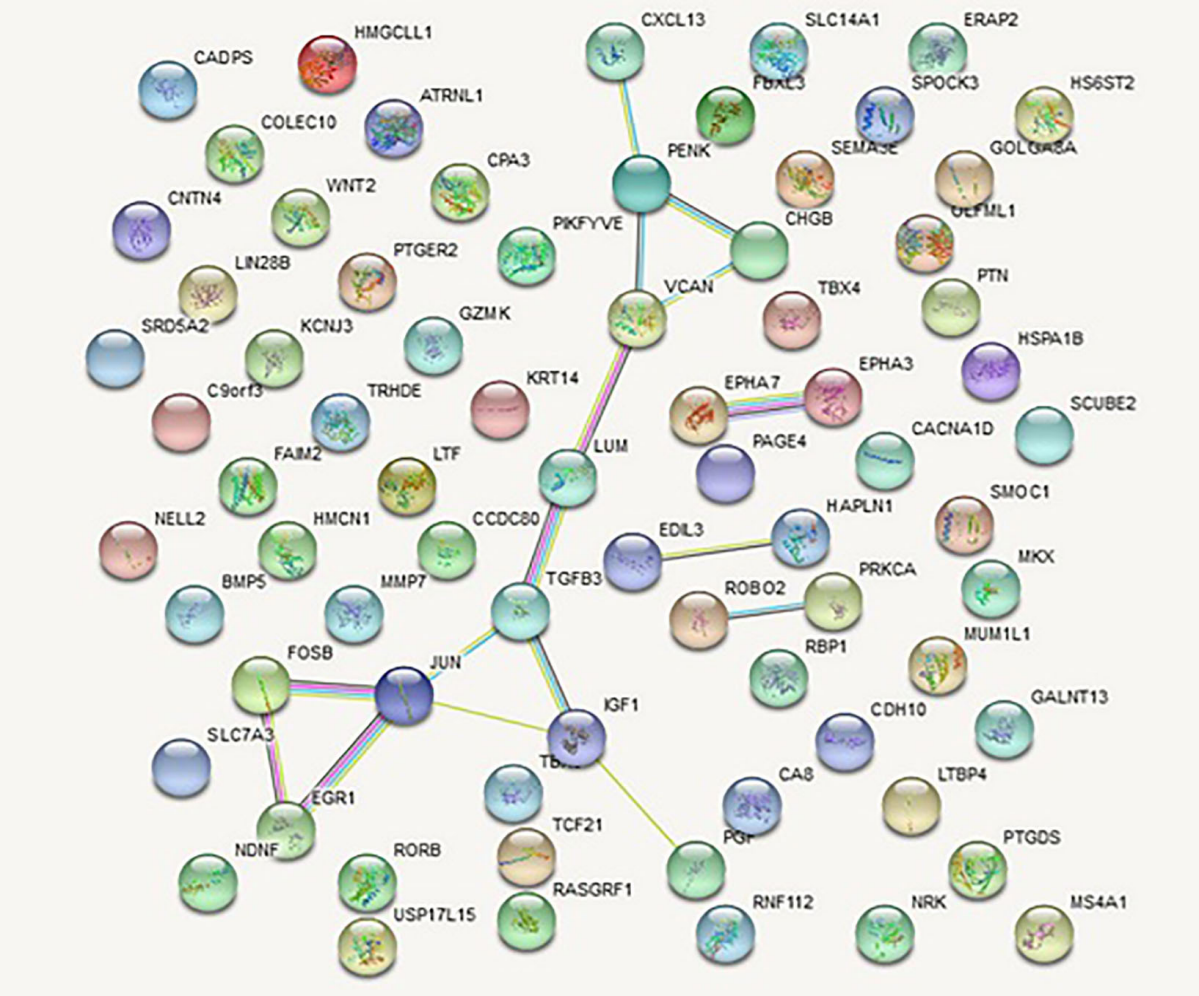
Protein-protein interaction network of up-regulated genes. Spheres represent the nodes and the lines represent the interactions

**Figure 2:**
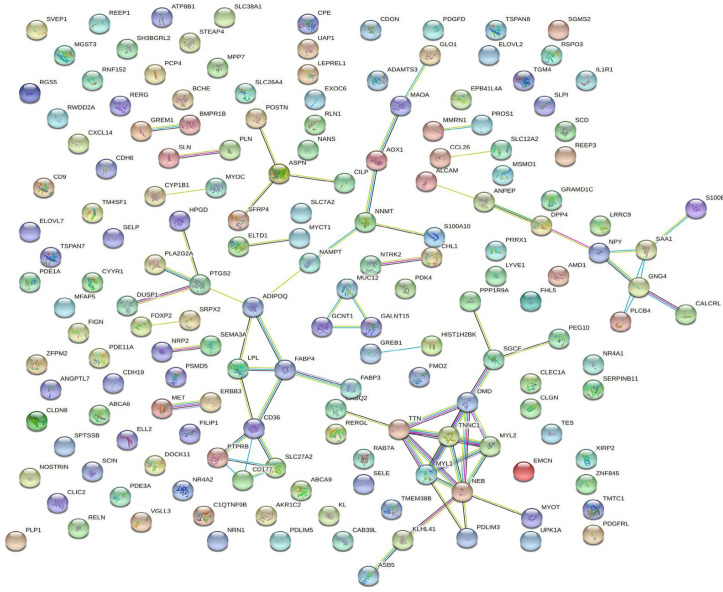
Protein-protein interaction network of down-regulated genes. Spheres represent the nodes and the lines represent the interactions

The DEGs obtained in the previous step were uploaded to the STRING database to examine their protein-protein interactions and a PPI network for both up-regulated and down-regulated genes was constructed and visualized using Cytoscape (Fig. 1 & 2). The PPI network for up-regulated and down-regulated genes consisted of 19 and 70 nodes with the clustering coefficient values of 0.424 and 0.254, respectively (Table 1). Although the up-regulated PPI network contains a lower number of nodes as compared to the down-regulated network, it displays a greater clustering coefficient indicating that it is more densely connected internally (Table 1).

**Table 1:** Topological parameters of PPI networks

**Topology parameters**	**Values of up-regulated ** **PPI network**	**Values of down-regulated PPI network**
Number of nodes	19	70
Number of edges	17	73
Clustering coefficient	0.424	0.254
Network density	0.236	0.144
Characteristic path length	2.891	3.627
Average number of neighbours	2.364	2.444

The networks were analyzed using NetworkAnalyzer and topological parameters were calculated. The topological analysis of the up-regulated network yielded top 5 nodes (degree >=3) represented as Transcription factor AP-1 (JUN), Versican core protein (VCAN), Proenkephalin (PENK), Insulin like growth factor 1 (IGF1) and Transforming growth factor beta 3 (TGFB3). These genes are enlisted in Table 2. Out of these nodes, Transcription factor AP-1 displays the highest degree while TGFB3 has the highest values of both centrality measures (Table 2). For the down-regulated network, top 12 nodes (degree>= 4) were identified as Adiponectin (ADIPOQ), fatty acid binding protein 4 (FABP4), prostaglandin-endoperoxide synthase 2 (PTGS2), Guanine nucleotide-binding protein subunit 4 (GNG4), Titin (TTN), Platelet glycoprotein 4 (CD36), Dystrophin (DMD), Myosin regulatory light chain 2 (MYL2), Troponin C1 (TNNC1), Myosin regulatory light chain 1 (MYL1), Nebulin (NEB) and Serum amyloid A1 (SAA1). Table 3 shows the list of top 12 nodes obtained from the down-regulated network. As can be seen from Table 3, values of degree suggest that the genes TTN, DMD, MYL1 & NEB show greater number of interactions in the network. ADIPOQ and NEB have the highest betweenness centrality and closeness centrality, respectively (Table 3). Combining all these top nodes together, 17 nodes were considered as hub genes.

**Table 2:** List of top nodes obtained from Up-regulated PPI network

**Serial number**	**Symbol**	**Name**	**Degree**	**Betweenness Centrality**	**Closeness Centrality**
1	JUN	Transcription factor AP-1	4	0.355556	0.416667
2	VCAN	Versican core protein	3	0.466667	0.4
3	PENK	Proenkephalin	3	0.2	0.322581
4	IGF1	Insulin like growth factor 1	3	0.2	0.4
5	TGB3	Transforming growth factor beta 3	3	0.555556	0.47619

**Table 3:** List of top nodes obtained from Down-regulated PPI network

**Serial number**	**Symbol**	**Name**	**Degree**	**Betweenness Centrality**	**Closeness Centrality**
1	ADIPOQ	Adiponectin	4	0.691176	0.425
2	FABP4	Fatty acid binding protein 4	4	0.279412	0.377778
3	PTGS2	Prostaglandin endoperoxide synthase 2	4	0.330882	0.34
4	GNG4	Guanine nucleotide binding protein subunit 4	4	0.321429	0.533333
5	TTN	Titin	6	0.153846	0.565217
6	CD36	Platelet glycoprotein 4	5	0.308824	0.314815
7	DMD	Dystrophin	6	0.384615	0.619048
8	MYL2	Myosin regulatory light chain 2	5	0	0.541667
9	TNNC1	Troponin C1	5	0	0.541667
10	MYL1	Myosin regulatory light chain 1	6	0.051282	0.565217
11	NEB	Nebulin	8	0.461538	0.65
12	SAA1	Serum amyloid A1	4	0.321429	0.533333

The hub genes identified in the previous step were analyzed for their co-expression with other genes using the FpClass tool. Top genes were selected using the criteria of network topology score of more than or equal to 0.70. Twenty one genes were found to be the top scoring predicted partners as can be seen in the Table 4. Thus, we obtained 17 hub genes from PPI network analysis and 21 hub genes from co-expression analysis, making it a total of 38.

**Table 4:** Top scoring predicted partners found by Fpclass

**Serial number**	**Query ID**	**Predicted Partner Symbol**	**Gene Co-expression Score**	**Network topology score**
1	TTN	MYPN	0	0.8423
2	VCAN	CCBP2	0.0262	0.7991
3	NEB	ANKRD23	0.0017	0.7813
4	JUN	HIF1A	0.0465	0.7809
5	JUN	DAXX	0.0207	0.7802
6	JUN	HSP90AA1	0.0271	0.7772
7	JUN	TP73	0.0136	0.7647
8	JUN	PML	0.0212	0.7645
9	JUN	PTPN1	0.0144	0.7609
10	JUN	PPARG	0.0231	0.7604
11	JUN	BTRC	0.0098	0.7575
12	JUN	CCND1	0.0214	0.7562
13	JUN	AHR	0.0744	0.752
14	JUN	SRC	0.016	0.751
15	JUN	HSPA8	0.0218	0.7507
16	VCAN	CCR4	0.0148	0.7503
17	TTN	SMURF2	0	0.7471
18	MYL2	ANKRD44	0.0014	0.7321
19	TTN	DES	0	0.7148
20	DMD	ABCA1	0.0358	0.7107
21	NEB	TRIM63	0.003	0.7031

The 38 hub genes were subjected to pathway analysis considering GO term as well as KEGG pathways. This analysis yielded 69 pathways in total. We found seven pathways enriched with hub genes such as cyclin D1 (CCND1), activator protein-1 (AP-1), prostaglandin-endoperoxide synthase 2 (PTGS2), adiponectin (ADIPOQ), SRC proto-oncogene (SRC), platelet glycoprotein 4 (CD36), transforming growth factor beta 3 (TGFB3), insulin like growth factor 1 (IGF1), hypoxia inducible factor 1-alpha (HIF1A), peroxisome proliferator activated receptor gamma (PPARG), troponin C1 (TNNC1). Table 5 provides a list of these pathways along with the parameters such as p value that indicates the significance of the pathway found. In Table 5, number of non-redundant genes and % associated genes represent the number of genes involved in a specific pathway while associated genes column gives the names of the hub genes found. As can be seen from Table 5, pathways enriched with hub genes, i.e. having>6 non-redundant genes were TGF-beta signaling pathway, Interleukin-4 and Interleukin-13 signaling, regulation of smooth muscle cell proliferation, smooth muscle cell proliferation, muscle filament sliding, striated muscle contraction pathway, dilated cardiomyopathy, hypertrophic cardiomyopathy. Among these, the striated muscle contraction and muscle filament sliding pathways are predominantly enriched with down-regulated hub genes whereas the TGF-beta signaling pathway mainly contains up-regulated hub genes (Table 5).

This was followed by identification of transcription factors (TFs) associated with the hub genes. We found 6 TFs - Zinc finger protein snai1, Steroidogenic factor 1, Aryl hydrocarbon receptor, E2F transcription factor 1, Nuclear factor of activated T-cells 1 and Interleukin enhancer-binding factor (Table 6). The most significant TF SNAI1 was found to interact with 6 hub genes (Table 6).

**Table 5:** Pathways enriched with hub genes

**Serial number**	**Pathway**	**P-Value**	**% Associated genes**	**Non Redundant genes**	**Associated genes**
1	Smooth muscle cell proliferation	4.60E-07	4.05	6	ADIPOQ, IGF1, JUN, PPARG, PTGS2, TGFB3
2	Hypertrophic cardiomyopathy	4.13E-10	7.78	7	DES, DMD, IGF1, MYL2, TGFB3, TNNC1, TTN
3	Dilated cardiomyopathy	6.53E-10	7.29	7	DES, DMD, IGF1, MYL2, TGFB3, TNNC1, TTN
4	Striated Muscle Contraction	4.97E-13	19.44	7	DES, DMD,MYL1, MYL2, NEB, TNNC1, TTN
5	Muscle filament sliding	9.13E-13	17.95	7	DES, DMD,MYL1, MYL2, NEB, TNNC1, TTN
6	Interleukin-4 and Interleukin-13 signaling	1.50E-09	6.48	7	CCND1, CD36, HIF1A, HSP90AA1, HSPA8, PTGS2, SAA1
7	TGF-beta Signaling Pathway	2.16E-07	4.51	6	BTRC, CCND1, JUN, PML, SMURF2, SRC

**Table 6:** Transcription factors associated with hub genes

**Transcription factor**	**Symbol**	**Interacting genes**	**P-Value**
Zinc finger protein snai1	SNAI1	PPARG, PTGS2, ADIPOQ, SRC, IGF1, HIF1A	0.173007
Steroidogenic factor 1	NR5A1	PPARG, JUN, ADIPOQ	0.134746
Aryl hydrocarbon receptor	AHR	PPARG, CCND1, JUN, PTGS2, SRC	0.128163
E2F transcription factor 1	E2F1	IGF1	0.115042
T-cells 1	1		
Interleukin enhancer-binding factor	ILF2	HIF1A, JUN	0.105026

**Figure 3:**
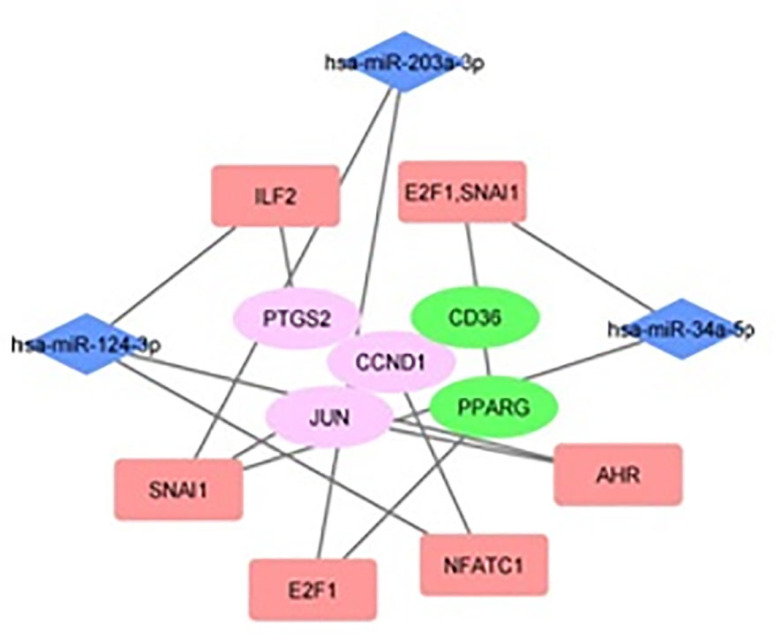
The transcription regulatory network showing the hub genes connected to the associated transcriptional factors (TFs) and the correlated miRNAs to the TFs in BPH. The central bigger nodes represent the hub genes; the rectangular boxes represent the TFs and are coloured in peach; the blue colored diamond shapes depict the miRNAs

Next step involved investigating miRNA linked with the TFs. As a result, we identified a total of 3 common miRNAs which were found to be connected to at least two of the TFs (Table 7). The miRNA hsa-miR-124-3p was the most prominent as it is specific for 3 TFs (Table 7). A transcription factor regulatory network was built as shown in the Figure (fig. 3). This network connects miRNAs to their target TFs that are further linked to their associated hub genes (Fig. 3). Two miRNAs - hsa-miR-203a-3p and hsa-miR-34a-5p target the transcription factor SNAI1 which is associated with the maximum number of the hub genes (Table 6 & Fig. 3).

**Table 7:** miRNAs associated with the regulatory factors from miRTarbase

**miRTarbase ID**	**miRNA**	**Interacting transcriptional factors**
MIRT006039	hsa-miR-203a-3p	SNAI1, E2F1
MIRT052946	hsa-miR-34a-5p	E2F1, SNAI1
MIRT022452	hsa-miR-124-3p	ILF2, AHR, NFATC1

## DISCUSSION

Cyclin D1 belongs to the family of cyclins which play a key role in cell cycle progression. In the presence of appropriate stimuli, cyclin D1 acts on cyclin dependent kinases (CDK) 4 and 6 and causes the release of a transcription factor which in turn stimulates the expression of genes required for DNA replication and progression from the G1 to S phase. Cyclin D1 has been implicated to be involved in the process of mast cells infiltration into BPH tissues [19]. Specifically, mast cells have been suggested to induce benign prostatic hyperplasia epithelial cell proliferation via cyclin D1 pathway that involves transcription factors such as Janus family tyrosine kinase (JAK)-signal transducer and activator of transcription (STAT).

Another hub gene transcription factor activator protein-1 (AP-1) was found to be up-regulated in BPH samples as revealed by differential gene expression analysis. This is in accordance with previous studies that have reported an increased expression of AP-1 in patients suffering from progressive symptomatic BPH and require surgical intervention as compared to mildly symptomatic patients [20]. AP-1 protein is a member of the JUN transcription factors family that plays a crucial role in regulating the transcription of genes involved in the processes of cellular proliferation and inflammation. Overexpression of AP-1 has been observed in colorectal cancer, breast cancer and acute myeloid leukemia, thereby implicating its role in the development and progression of cancers [21]. Thus, AP-1 may represent a potential target that can be employed to target the underlying problems of inflammation and proliferation associated with BPH.

Adiponectin is an adipokine secreted from adipose tissues and exerts multiple properties including anti-proliferation, anti-inflammation, anti-apoptotic and anti-tumor [22]. It is known to activate fatty acid beta-oxidation and blocks glucose production in the liver. Lower levels of adiponectin in serum have been linked with a higher risk of benign prostatic hyperplasia [23]. In our study, the gene encoding adiponectin was found to be down-regulated in samples collected from BPH patients.

Hub genes encoding for myosin regulatory light chains 1 & 2 were found to be down-regulated in BPH patients. Previous studies have reported reduced expression of myosin light chains in detrusor smooth muscle from the rabbit model of partial urinary bladder outlet obstruction (PBOO) which is commonly observed in men suffering from BPH [24]. This indicates that altered expression of myosin in the urinary bladder may be responsible for the abnormal voiding behaviour encompassing LUTS in BPH patients. These results suggest that abnormal muscle contraction and relaxation may be corrected by targeting myosin in bladder smooth muscles.

An upregulated gene found in our study was VCAN that encodes for versican core protein, an important component of the extracellular matrix. Versican is a proteoglycan which plays a critical role in inflammation and tissue homeostasis. Sakko et al. have reported an increase in versican levels in prostate tumor cells isolated from BPH patients [25]. This increased production of versican was seen to be mediated through transforming growth factor beta1 (TGFB1). Interestingly, our study has found the involvement of six of the up-regulated hub genes in the TGF beta signaling pathway, confirming that increased expression of versican is driven through transforming growth factor. In addition, the role of versican has been elucidated in lung disorders including chronic obstructive pulmonary disease and asthma which showed an upregulation of the protein [26]. Proteoglycans including versican are thought to be involved in tissue remodelling, thereby highlighting the potential of versican as a therapeutic drug target.

Another hub gene Insulin like growth factor 1 (IGF1) was found to be up-regulated. This is in accordance with previous studies which have reported increased expression of IGF1 in BPH patients with greater prostate size [27]. Another study has shown that silencing of IGF1 gene in epithelial cells of the prostate leads to reduced cell proliferation [28]. These findings indicate that IGF1 can be potentially targeted as an effective therapeutic strategy to tackle BPH.

The hub gene hypoxia inducible factor 1-alpha (HIF1A) is a transcription factor that controls the expression of hypoxia-induced genes such as glycolytic enzymes, cell growth factors and erythropoietin. In addition, HIF1A also induces epithelial–mesenchymal transition (EMT) that involves conversion of epithelial cells into mesenchymal phenotype [29]. EMT has been found to be associated with tumor progression and tissue remodeling. Specifically, HIF1A activates the expression of epithelial–mesenchymal transition-promoting transcription factors such as Snail, thereby leading to EMT. Interestingly, we found a close association between HIF1A and Snail transcription factor, further confirming the role of HIF1A in mediating EMT. The role of HIF1A has been elucidated in previous studies. It has been reported to cause testosterone-induced hyperplasia in the prostate gland of rats [30]. Another study has revealed that under inflammatory conditions, the prostate gland secretes cytokines which stimulate the expression of HIF1A that leads to prostate remodeling through the process of EMT [31]. Thus, results from previous studies and our study strongly suggest that HIF1A poses a great potential to control the tumor progression in BPH.

Peroxisome proliferator activated receptor gamma (PPARG) belongs to the nuclear receptor superfamily that comprises ligand-activated transcription factors. Nuclear receptors are considered as the second largest class of therapeutic drug targets. PPARG serves as a key regulator of beta-oxidation of fatty acids, adipocyte differentiation and glucose homeostasis [32]. *In vivo* studies have demonstrated that expression of PPARG promotes adipogenesis in fibroblast cell lines [33]. This finding has also been supported by evidence obtained from mice lacking PPARG gene [34]. We found peroxisome proliferator activated receptor gamma (PPARG) to co-express with another hub gene i.e JUN which showed 2-fold up-regulation in BPH patients. This finding correlates well with the fact that obesity is one of the most common co-morbidities observed in BPH patients [35]. Hence, therapeutic alterations in PPARG activity seem to be an effective treatment strategy to relieve BPH and its co-morbidities.

Pathway enrichment analysis revealed that genes involved in pathways such as striated muscle contraction and muscle filament sliding pathways were down-regulated. This may be one of the possible causes of voiding symptoms observed in BPH patients. One of the most effective medical treatments for BPH is to induce prostate smooth muscle relaxation. Thus then genes encoding for dystrophin (DMD), myosin regulatory light chain 1 (MYL1), myosin regulatory light chain 2 (MYL2), nebulin (NEB), troponin C1 (TNNC1), titin (TTN) participate in muscle contraction and muscle sliding pathways. These genes are good candidates to develop therapeutic strategies aiming at relieving LUTS which is a major concern in BPH disorder.

The TFs found to primarily regulate the expression of the hub genes were Zinc finger protein snai1 (SNAI1), Aryl hydrocarbon receptor (AHR), Nuclear factor of activated T-cells 1 (NFATC1) (Table 6). These were mainly associated with hub genes PPARG, SRC, IGF1. One of the common pathways among these genes is the transforming growth factor-β (TGF-β) signaling pathway. A study has earlier demonstrated that abnormal activation of TGF-β resulted in mobilization of stromal stem cells which ultimately recruited to the prostate tissues leading to its overgrowth in BPH patients [36]. In accordance with this, we found that the hub genes involved in the TGF-β signaling pathway showed 2-fold greater expression in men suffering from BPH. These results combined together suggest that inhibiting the activity of TGF-β could serve as an effective therapeutic intervention for this disease. 

Another important pathway is regulation of smooth muscle cell proliferation. One of the well-tested therapeutic strategies to treat LUTS in BPH patients is to relax prostate smooth muscles [37, 38]. They have mainly targeted alpha(1A)-adrenergic receptors and phosphodiesterase 5 (PDE5). But these treatments have been shown to cause some side effects such as sexual dysfunction. So, alternative targets should be considered to tackle the problem of LUTS. Our study has found six genes which play key roles in the regulation of smooth muscle cell proliferation. These include SRC proto-oncogene (SRC), Beta-Transducin Repeat Containing E3 Ubiquitin Protein Ligase (BTRC), cyclin D1 (CCND1), transcription factor activator protein-1 (AP-1), Promyelocytic leukemia protein (PML), SMAD Specific E3 Ubiquitin Protein Ligase 2 (SMURF2) which could be targeted to provide relief from voiding symptoms associated with BPH.

Overall, our study highlighted various biological functions of hub genes and their roles in signaling pathways which correlate with the symptoms and common co-morbidities of BPH. Thus, a detailed understanding of functions of hub genes provided important insights into the disease pathophysiology and such information can be utilized to develop effective medical treatment strategies for BPH.

## Conflict of Interest

All authors declare no conflict of interest.
